# Comparison of the Outcomes of Cadexomer Iodine and Povidone-Iodine Ointments in Wound Management

**DOI:** 10.7759/cureus.24667

**Published:** 2022-05-02

**Authors:** Shubham Gupta, Raju K Shinde, Sangita Shinde

**Affiliations:** 1 General Surgery, Datta Meghe Institute of Medical Sciences, Deemed to be University, Wardha, IND; 2 Pharmacology, Datta Meghe Institute of Medical Sciences, Deemed to be University, Wardha, IND

**Keywords:** povidone-iodine, chronic venous leg ulcers, traumatic ulcer, infective ulcer, diabetic ulcer, cadexomer iodine

## Abstract

Background

In this era of upcoming newer formulations of topical ointments in the market, selecting an appropriate topical ointment for managing ulcers is challenging with regards to granulation tissue promotion, ulcer size reduction, and decrease in the amount of discharge from the ulcer. This study compares the outcomes of two topical iodine formulations, namely, cadexomer iodine ointment (0.9%) and povidone-iodine ointment (5%), for the management of various types of ulcers.

Methodology

This prospective, interventional study was conducted in a tertiary care hospital. After screening, 40 patients with ulcers (venous, arterial, diabetic, traumatic, infective) were subjected to simple randomization based on computer-generated random numbers at a ratio of 1:1 for the application of cadexomer (n = 20) (Group A) and povidone-iodine ointment (n = 20) (Group B). Selected patients were subjected to broad-spectrum antibiotics on admission and then shifted to special antibiotics based on the culture and sensitivity report of the wound. The application of these two formulations was done in three settings, each lasting 48 hours. The efficacy of these two formulations was assessed based on the following three parameters: (a) the percentage of granulation tissue promotion, (b) wound size reduction, and (c) decline in wound discharge. The study groups were compared using an unpaired t-test, while the association among the study groups was performed using Fishers’ test, Student’s t-test, and chi-square test. P-values less than 0.05 were considered significant.

Results

Significant improvement (p < 0.05) in granulation tissue was observed with cadexomer ointment application compared to povidone-iodine ointment. Statistically significant reductions in ulcer size and discharge from ulcers were seen in both groups; however, clinically, cadexomer ointment was found to be more effective compared to povidone-iodine ointment in reducing ulcer size as well as in reducing the amount of discharge from ulcers.

Conclusions

Cadexomer iodine ointment proved to be better than povidone-iodine ointment in the management of ulcers regarding the percentage of granulation tissue promotion, ulcer size reduction, and decrease in the amount of discharge from ulcers.

## Introduction

Wound care is a serious public health issue that affects a large number of people with various types of wounds and costs a large number of resources. Wound care places a considerable burden on patients, healthcare providers, and the entire healthcare system [1].

Ulcer management is challenging as it leads to pain, frequent hospitalization, disfigurement, disability, prolonged rehabilitation, loss of income, loss of job, and an enormous financial burden to the patient. Chronic wounds are a hidden epidemic that impacts numerous people globally [2]. In advanced nations, it is expected that 1-2% of the population may suffer from a chronic wound at some point in their lives [3]. Moreover, 60% of chronic wounds and 6% of acute wounds have been shown to have biofilms, and eradicating the residual bacteria is challenging [4,5]. Therefore, for effective wound healing, antiseptics should ideally target both biofilm production and inflammation.

Appropriate wound care products should preserve normal tissues, prevent infection, and not hinder the normal healing process of the wound. The key to success is complete wound healing which is accomplished by meticulous wound care and optimization of the wound healing capacity.

The wound bed must be properly vascularized, free of necrotic tissues, infection-free, and moist to ensure good healing. In general, only a few antibacterial compounds are recommended for wound treatment. Of these, povidone-iodine has remained the most widely used [6].

Cadexomer iodine is one such formulation that has been documented to help minimize and eliminate these barriers to the recovery and repair of the tissues. This treatment contains hydrophilic beads of cadexomer starch (size ranging from 0.1 to 0.3 mm) which is biodegradable and spherical in shape and contains 0.9% w/w iodine. It is marketed as gel/ointment, powder, and dressing foams. Cadexomer iodine exhibits a dual mode of action that combines physical and antibacterial properties to handle various impediments to healing, including excessive exudate, bioburden, and slough [7]. Cadexomer beads absorb exudative fluid up to seven times their weight, facilitating excellent wound exudate management. Cadexomer beads physically inflate when they encounter exudative fluid, allowing for sustainable availability of iodine for killing biofilm and bacteria inside the dressing for up to three days (or 72 hours) with minimal toxicity. Excess pus, slough, exudate and bioburden, and necrotic debris, including biofilm, are all factors that hinder wound healing. When these barriers are effectively managed or removed, a wound habitat favorable for healing is generated, which allows further wound healing [8-10].

The most often used iodophor, povidone-iodine, has numerous features that make it ideal for wound healing, including a broad antibacterial range, lack of resistance, efficacy against biofilms, good tolerability, and influence on excessive inflammation. In a study by Murdoch and Lagan [11], cadexomer iodine was particularly efficient in chronic wounds, while povidone-iodine was more beneficial in infected acute wounds.

Hence, this study was conducted at our tertiary care center to compare the outcomes of povidone-iodine and cadexomer iodine ointments in the management of wounds.

## Materials and methods

This hospital-based, prospective, interventional study was conducted among 40 patients to compare the outcomes of cadexomer iodine and povidone-iodine ointments in wound management.

Study design

A hospital-based, prospective, interventional study was carried out from September 2019 to December 2021 after obtaining approval from the Institutional Ethical Committee (IEC) at a tertiary care center in the Department of General Surgery, Acharya Vinoba Bhave Rural Hospital (AVBRH), Sawangi, Meghe, Wardha.

Study population

All patients with acute or chronic wounds measuring more than 25 cm^2^ who were admitted to AVBRH, a tertiary care hospital, after a valid, written and informed consent and who fulfilled the inclusion criteria comprised the study population. The sample size was calculated using the following formula: n = [z2p(1-p)]/d2 = 18.9. In total, 20 patients in each group were needed to detect a significant difference; hence, a sample size of 40 patients was selected for the study.

All patients were enrolled and grouped into two groups: Group A (n = 20): patients with wounds/ulcers subjected to cadexomer iodine ointment application. Group B (n = 20): patients with wounds/ulcers subjected to povidone-iodine ointment application.

Inclusion and exclusion criteria

Patients of both genders (male and female) with ages ranging between 15 and 90 years were considered for inclusion in the study. Patients with venous leg ulcers, diabetic foot ulcers, and pressure ulcers with adequate arterial blood supply assessed using color Doppler, as well as all patients with acute or chronic wounds measuring more than 25 cm^2^ were included in this study.

Patients on immunosuppressing, corticosteroid, and anti-cancer drugs, those with severe malnutrition (serum albumin <3.0 g/dL or total protein <6.5 g/dL), those with renal dysfunction (serum creatinine >3.0 mg/dL), those with hepatic dysfunction, and those with thyroid dysfunction were excluded from the study.

Methodology

**Figure 1:**
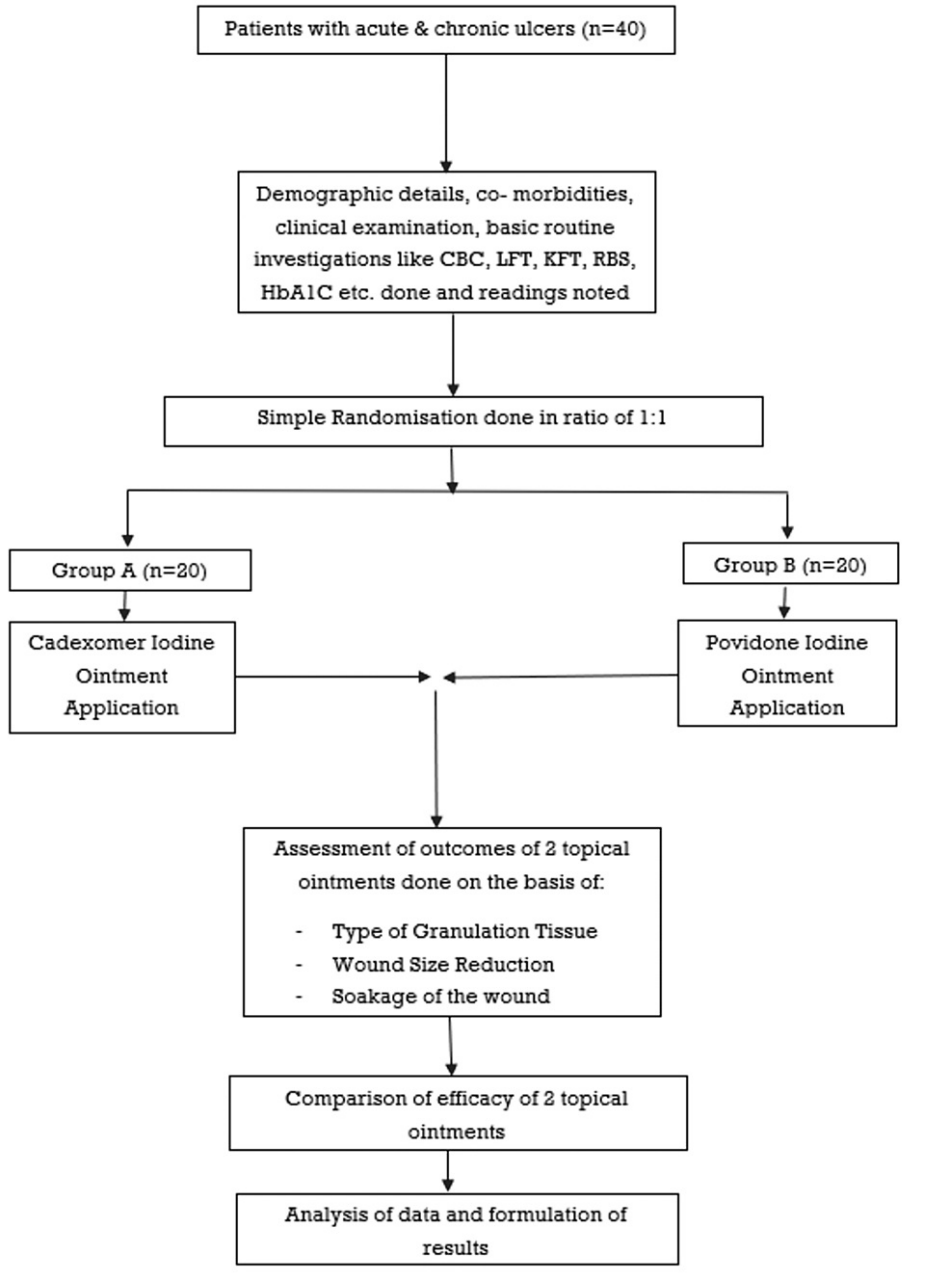
Flowchart of the methodology. CBC: complete blood count; LFT: liver function test; KFT: kidney function test; RBS: random blood sugar; HbA1c: glycated hemoglobin

Eligible and consenting patients were randomly assigned to one of two treatments using a simple randomization procedure based on computer-generated random numbers at a ratio of 1:1. Figure 1 shows a flowchart of the methodology adopted in this study.

Calculation of the Size of the Wound

The surface area [12] was calculated by placing an acetate sheet over the wound and tracing the wound border with a permanent pen. The area of the wound was calculated by counting the complete and half or more than half squares within the wound border taking it as 1 cm^2^.

In our study, we used two formulations of iodine, i.e., cadexomer iodine ointment containing 0.9% w/w iodine and povidone-iodine ointment containing 5% w/w iodine.

**Figure 2:**
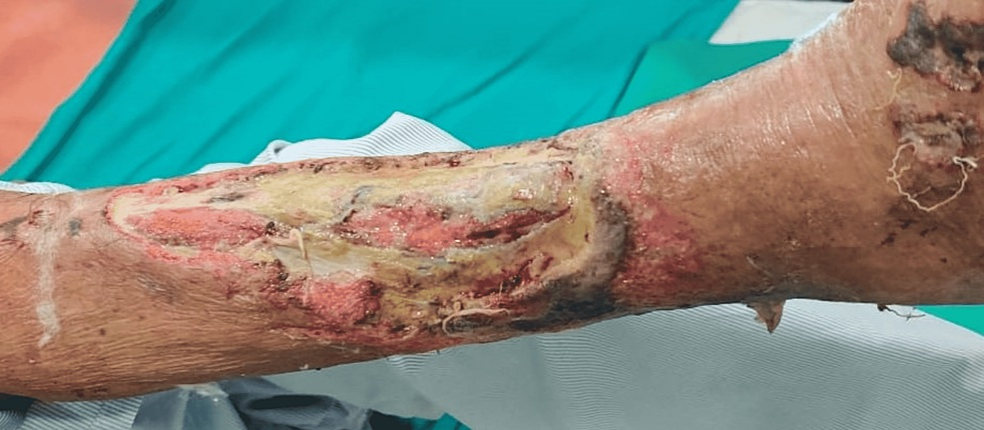
The initial wound image of a traumatic leg ulcer.

The cadexomer iodine was applied among group A patients and povidone-iodine ointment was applied among group B patients in three sittings of 48 hours (two days). The ointments were applied to a minimum of 3 mm thickness across the wound surface, following the configuration of the ulcer (Figure 2).

Assessment Criterion and Assessment Protocol

At the end of the study, both groups were assessed for the percentage of decrease in wound size, type of granulation tissue over the wound, and amount of soakage of the wound.

**Figure 3:**
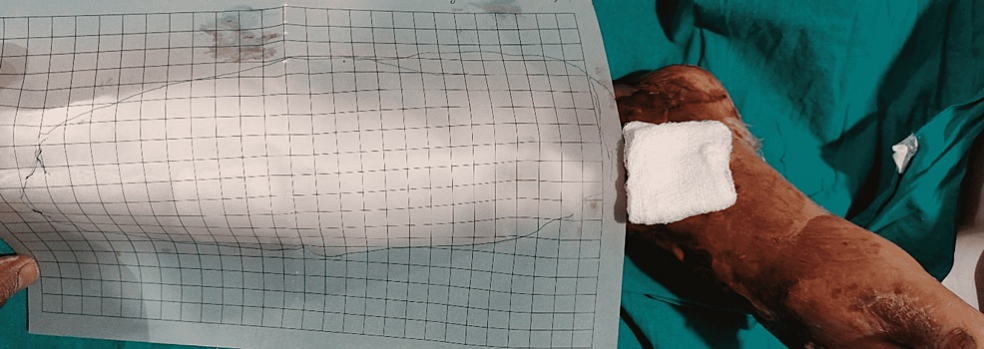
Measuring the wound size using tracing paper.

The percentage of wound size reduction was calculated based on the decrease in the area of the wound. Ulcer size was measured at the end of each of the three sittings (Figure 3), and the area was then calculated by counting the total number of complete and half or more than half squares within the wound border, taking each square size as an area of 1 cm^2^.

We categorized wounds into the following three categories based on the area of the wound: Small: wounds having a total surface area of 1-10 cm^2^; medium: wounds having a total surface area of 11-20 cm^2^; large: wounds having a total surface area of 21-30 cm^2^.

The type of granulation tissue [13] of the wound was classified into the following three: Healthy granulation: pink in color composed of vascularized connective tissue; unhealthy/pale granulation: dark red in color often bleeds on contact, may indicate the presence of an infection; Slough: dead tissue usually cream or yellow in color.

On a similar note, wounds were also classified on the basis of the amount of soakage or exudate [14] (percentage of dressing soaked in 24 hours): Small: discharge involving less than 25% of dressing; Moderate: drainage involving 25-75% of dressing; Copious: drainage involving more than 75% of dressing.

**Figure 4:**
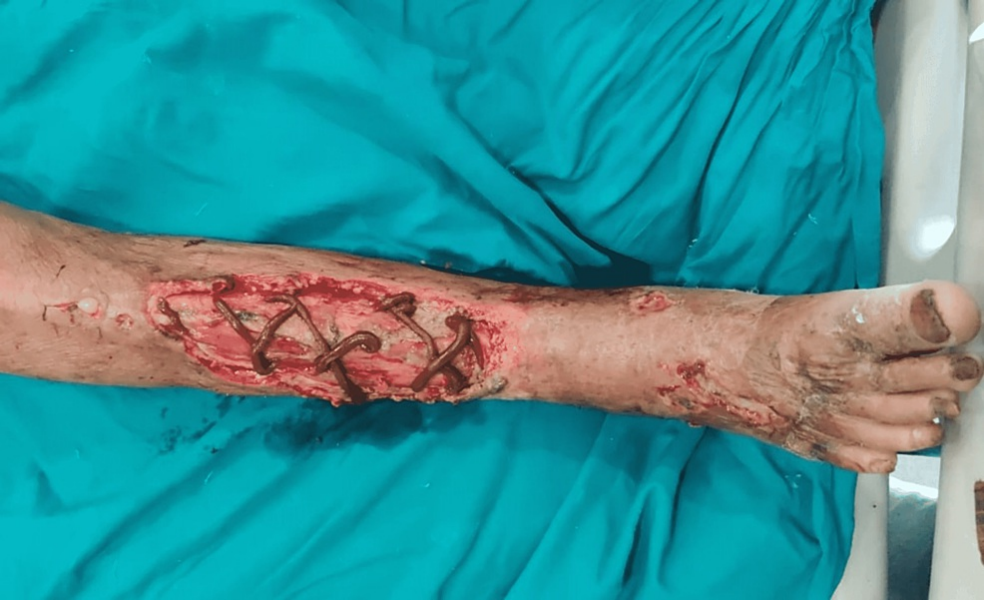
Application of iodine ointment over the wound.

**Figure 5:**
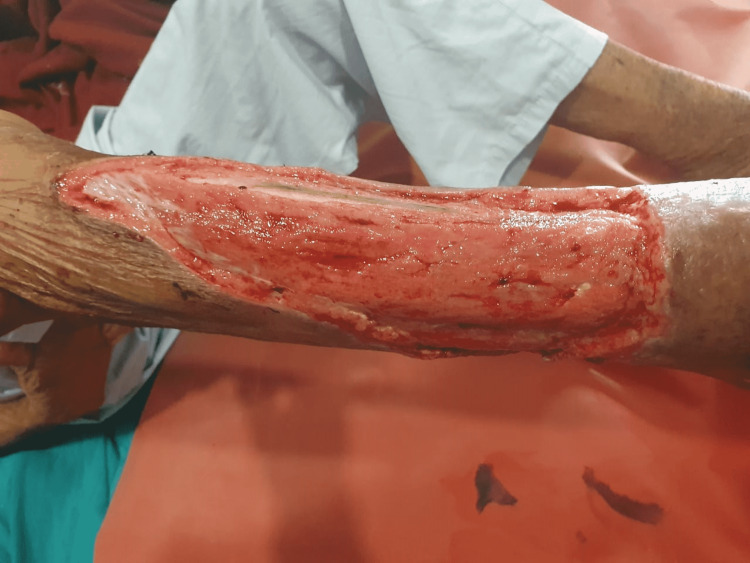
Wound after three sittings of iodine ointment application.

Figure 4 shows the application of iodine ointment over a wound, and Figure 5 shows the wound after ointment application for three sittings.

Statistical analysis

Quantitative data are presented as mean and standard deviation. Comparison among the study groups was done using an unpaired t-test as per the results of the normality test. Qualitative data are presented as frequency and percentage. The association among the study groups was assessed using Fisher’s test, Student’s t-test, and chi-square test. P-values of less than 0.05 were considered significant. Appropriate statistical software, including but not restricted to MS Excel and SPSS version 20 (IBM Corp., Armonk, NY, USA), was used for statistical analysis. Graphical representation was in MS Excel 2010.

## Results

Comparison of initial wound size with wound size after first, second, and third sittings in group A

**Figure 6:**
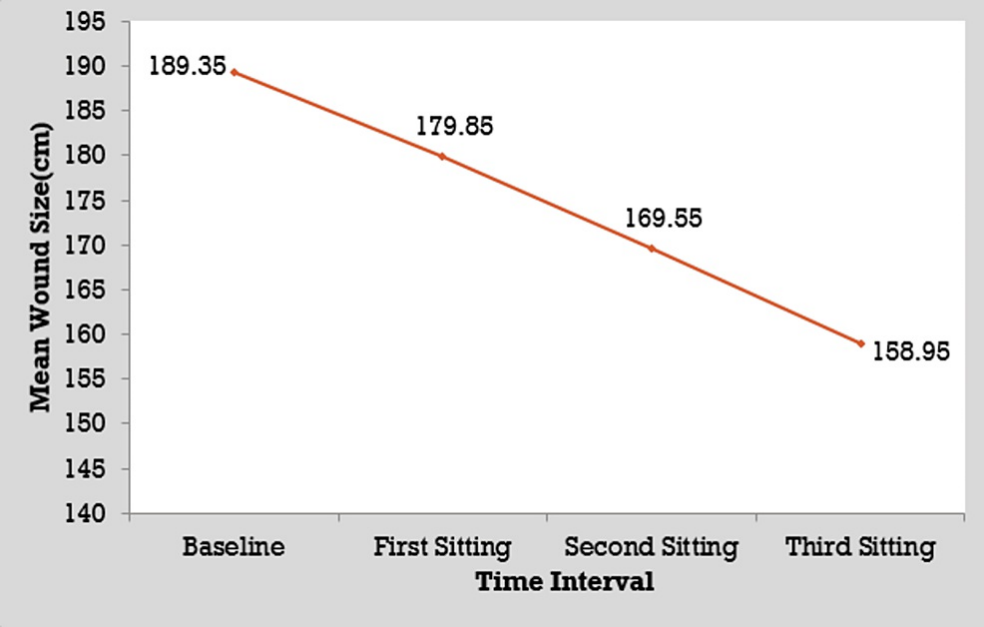
Comparison of the initial wound size with wound size after first, second, and third sittings in group A.

The mean wound size at baseline in group A patients was 189.35 ± 58.72 cm^2^ which decreased significantly to 179.85 ± 58.61 cm^2^ by the first sitting, 169.55 ± 58.69 cm^2^ by the second sitting, and 158.95 ± 58.43 cm^2^ by the third sitting. There was a significant difference in wound area during the follow-up period according to the analysis of variance (ANOVA) test (p < 0.05) (Figure 6).

Comparison among all sittings and with baseline in group A

The mean difference between baseline and the first sitting was 9.50 ± 2.48 cm^2^, while the mean difference between baseline and the second sitting and baseline and the third sitting was 19.80 ± 3.41 cm^2^ and 30.40 ± 4.27 cm^2^, respectively. The mean difference between the first and second sittings was 10.30 ± 2.57 cm^2^, while the mean difference between the first and third sittings was 20.90 ± 3.94 cm^2^. The mean difference between the second and third sittings was 10.60 ± 2.47 cm^2^. The differences between the baseline and all sittings were significant as per the Student’s t-test (p < 0.05).

Comparison of the initial wound size with wound size after first, second, and third sittings in group B

**Figure 7:**
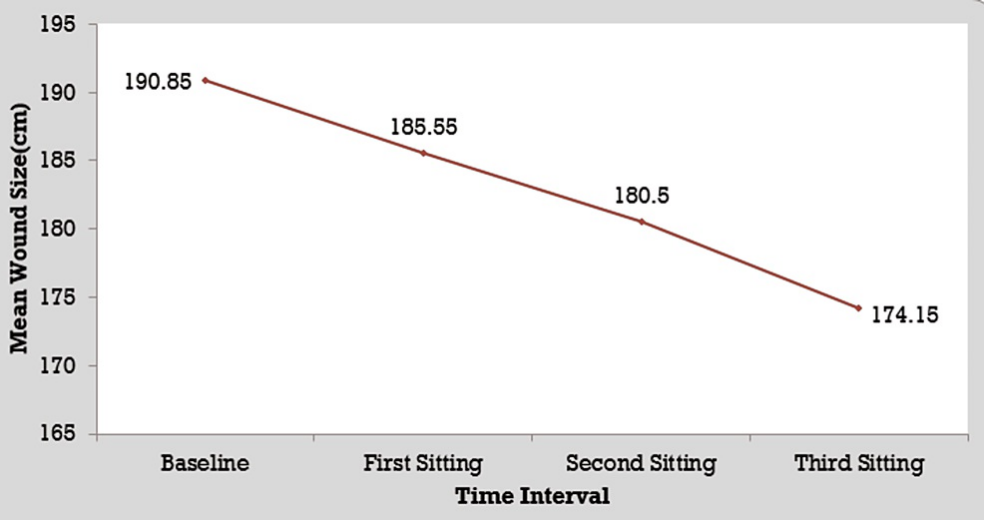
Comparison of the initial wound size with wound size after first, second, and third sittings in group B.

The mean wound size at baseline in group B patients was 190.85 ± 76.28 cm^2^ which decreased significantly to 185.55 ± 76.61 cm^2^ by the first sitting, 180.50 ± 76.47 cm^2^ by the second sitting, and 174.15 ± 77.06 cm^2^ by the third sitting. There was a significant difference in wound area during the follow-up period as per the ANOVA test (p < 0.05) (Figure 7).

Comparison among all sittings and with baseline in group B

The mean difference between baseline and the first sitting was 5.30 ± 2.25 cm^2^, while the mean difference between baseline and the second sitting and baseline and the third sitting was 10.35 ± 3.08 cm^2^ and 16.70 ± 3.81 cm^2^, respectively. The mean difference between the first and second sittings was 5.05 ± 1.53 cm^2^, while the mean difference between the first and third sittings was 11.40 ± 2.21 cm^2^. The mean difference between the second and third sittings was 6.35 ± 1.49 cm^2^. The differences between the baseline and all sittings were significant as per the Student’s t-test (p < 0.05).

Comparison of granulation tissue at baseline with granulation tissue after first, second, and third sittings in group A

In total, 13 (65%) and seven (35%) patients in group A had mild and moderate slough, respectively, at baseline, while seven (35%) and 13 (65%) had mild slough and pale granulation, respectively, at the first sitting. Overall, seven (35%) and 13 (65%) patients had pale and healthy granulation, respectively, at the second sitting, while all patients had healthy granulation by the third sitting. There was a significant improvement in granulation tissue from baseline till the third sitting as per the chi-square test (p < 0.05).

Comparison of granulation tissue at baseline with granulation tissue after first, second, and third sittings in group B

In total, six (30%) and 14 (70%) patients in group B had moderate slough and pale granulation at baseline, first sitting, and second sitting. Overall, three (15%) patients had moderate slough while 10 (50%) and seven (35%) patients had pale and healthy granulation, respectively, by the third sitting. There was a significant improvement in granulation tissue from baseline till the third sitting as per the chi-square test (p < 0.05).

Comparison of granulation tissue during first, second, and third sittings between groups

**Figure 8:**
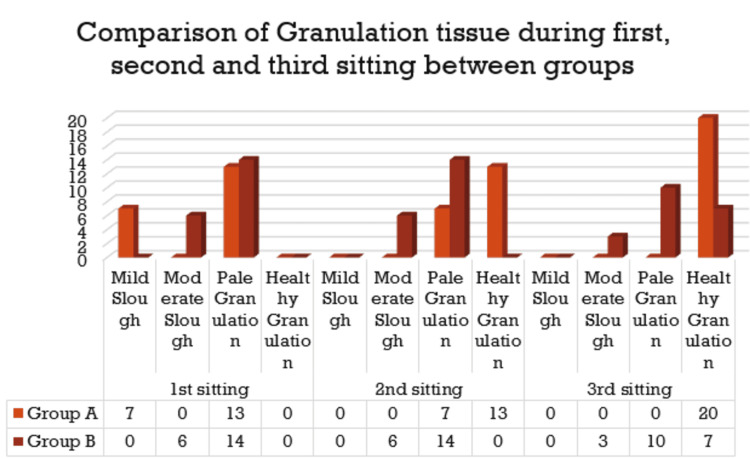
Comparison of granulation tissue during the first, second, and third sittings between groups.

There was a significantly higher number of patients in group A compared to group B who showed improvement in granulation tissue in all sittings as per the chi-square test (p < 0.05) (Figure 8).

Comparison of discharge at baseline with discharge after first, second, and third sittings in group A

In total, 13 (65%) and seven (35%) patients in group A had mild and moderate discharge, respectively, at baseline. All patients had mild discharge at the first, second, and third sittings. There was a significant improvement in discharge from baseline till the third sitting as per the chi-square test (p < 0.05).

Comparison of discharge at baseline with discharge after first, second, and third sittings in group B

In total, 14 (70%) and six (30%) patients in group B had mild and moderate discharge, respectively, at baseline, first, and second sitting. In the third sitting, 15 (75%) and three (15%) patients had mild and moderate discharge, respectively, while two (10%) patients had no discharge. There was a significant improvement in discharge from baseline till the third sitting as per the chi-square test (p < 0.05).

## Discussion

The microbicidal effects of iodine appear to include the suppression of essential bacterial cellular functions and structures, as well as the oxidation of nucleotides, fatty/amino acids, and cytosolic enzymes involved in the respiratory chain, causing them to become denatured and deactivated. Although cadexomer iodine has a higher moisture retention capacity (up to 6 mL/g of cadexomer iodine), the enhanced efficacy of cadexomer iodine dressings is not entirely due to variations in the water retention capacity of the dressing materials used.

Because of its broad antibacterial range and effectiveness in various types of wounds, povidone-iodine has been used as a topical treatment since the 1950s, and no resistance has been established to date [15]. Over the past couple of decades, there has been a surge in interest in using iodine to treat biofilms in chronic infected wounds.

In our study, the mean wound size at baseline in group A patients was 189.35 ± 58.72 cm^2^ which decreased significantly to 179.85 ± 58.61 cm^2^ by the first sitting, 169.55 ± 58.69 cm^2^ by the second sitting, and 158.95 ± 58.43 cm^2^ by the third sitting. There was a significant difference in wound area during the follow-up period as per the ANOVA test (p < 0.05). This is comparable to the study reported by Raju et al. [16].

In our study, the mean difference between the baseline and first sitting was 9.50 ± 2.48 cm^2^, while the mean difference between the baseline and second sitting and the baseline and third sitting was 19.80 ± 3.41 cm^2^ and 30.40 ± 4.27 cm^2^, respectively. The mean difference between the first and second sittings was 10.30 ± 2.57 cm^2^, while the mean difference between the first and third sittings was 20.90 ± 3.94 cm^2^. The mean difference between the second and third sittings was 10.60 ± 2.47 cm^2^. The differences among baseline and all sittings were significant as per the Student’s t-test (p < 0.05). Similar observations were noted in earlier studies [16,17].

In the present study, the mean wound size at baseline in group B patients was 190.85 ± 76.28 cm^2^, which decreased significantly to 185.55 ± 76.61 cm^2^ by the first sitting, 180.50 ± 76.47 cm^2^ by the second sitting, and 174.15 ± 77.06 cm^2^ by the third sitting. There was a significant difference in wound area during the follow-up period as per the ANOVA test (p < 0.05). The mean difference between the baseline and first sitting was 5.30 ± 2.25 cm^2^, while the mean difference between the baseline and second sitting and the baseline and third sitting was 10.35 ± 3.08 cm^2^ and 16.70 ± 3.81 cm^2^, respectively. The mean difference between the first and second sittings was 5.05 ± 1.53 cm^2^, while the mean difference between the first and third sittings was 11.40 ± 2.21 cm^2^. The mean difference between the second and third sittings was 6.35 ± 1.49 cm^2^. The differences among baseline and all sittings were significant as per the Student’s t-test (p < 0.05).

In our study, 13 (65%) and seven (35%) patients in Group A had mild and moderate slough, respectively, at baseline, while seven (35%) and 13 (65%) had mild slough and pale granulation, respectively, at the first sitting. Overall, seven (35%) and 13 (65%) patients had pale and healthy granulation, respectively, at the second, sitting while all patients had healthy granulation by the third sitting. There was a significant improvement in granulation tissue from baseline till the third sitting as per the chi-square test (p < 0.05).

It was observed in this study that six (30%) and 14 (70%) patients in group B had moderate slough and pale granulation at the baseline, first sitting, and 2nd sitting. Overall, three (15%) patients had moderate slough, while 10 (50%) and seven (35%) patients had pale and healthy granulation, respectively, by the third sitting. There was a significant improvement in granulation tissue from baseline till the third sitting as per the chi-square test (p < 0.05). There were a significantly higher number of patients in group A compared to group B who showed improvement in granulation tissue in all sittings as per the chi-square test (p < 0.05). This finding is similar to previous studies [17-20].

Cadexomer iodine, with its antibacterial and desloughing qualities, is recognized to eliminate the hurdle to healing [21]. The prolonged release of iodine provides broad-spectrum antibacterial action, while the unique cadexomer matrix provides desloughing action.

It was observed in our study that 13 (65%) and seven (35%) patients in group A had mild and moderate discharge, respectively, at baseline. All patients had mild discharge at the first, second, and third sittings. There was a significant improvement in discharge from baseline till the third sitting as per the chi-square test (p < 0.05).

In this study, 14 (70%) and six (30%) patients in group B had mild and moderate discharge, respectively, at the baseline, first sitting, and second sitting. In the third sitting, 15 (75%) and three (15%) patients had mild and moderate discharge, respectively, while two (10%) patients had no discharge. There was a significant improvement in discharge from baseline till the third sitting as per the chi-square test (p < 0.05). Similar observations were noted in the study by Raju et al. [16].

Despite differences in therapy, povidone-iodine ointment had a significantly lower proportion of reducing ulcer size and a significantly lower number of patients with effective wound healing than cadexomer iodine ointment.

Limitations and recommendations

This study had a very small sample size, and there is limited literature and research available on this topic of comparing the outcomes of cadexomer iodine and povidone-iodine ointments for wound management. Thus, we recommend more such studies and research among larger sample sizes to determine the efficacy and efficiency of cadexomer iodine ointment and its comparison with traditional topical formulations for wound care management.

## Conclusions

Cadexomer iodine ointment was found to be more effective for promoting healthy granulation tissue formation earlier than povidone-iodine ointment, leading to earlier preparation of wounds or ulcers for definitive therapy or intervention. With these potential benefits, cadexomer iodine stands out as another competent, reliable, and efficient therapeutic option for physicians and surgeons treating patients with infected wounds or ulcers. Both cadexomer iodine and povidone-iodine ointments are safe and efficient in the management of wounds or ulcers, potentially improving the quality of life of patients and lowering healthcare expenses.
